# Integrated metabolomics and metagenomics reveal divergent caecal metabolic signatures following commercial gut health interventions in broilers

**DOI:** 10.1186/s42523-026-00596-z

**Published:** 2026-07-04

**Authors:** Gladys Maria Pangga, Anne Richmond, Callie Hughes, Androniki Psifidi, Dong Xia, Damer Blake, Umer Zeeshan Ijaz, Ozan Gundogdu

**Affiliations:** 1 London School of Hygiene & Tropical Medicine, London, UK; 2 Pilgrim’s Europe Ltd, Craigavon, UK; 3 Royal Veterinary College, London, UK; 4 University of Glasgow, Glasgow, UK

**Keywords:** Untargeted metabolomics, Integrative omics, Poultry gut health, Coccidiosis, Ionophore, *Eimeria* vaccination

## Abstract

**Background:**

The intensification of food production systems highlights the need for poultry gut health strategies aligned with One Health goals. Central to this is a balanced gut microbiota, which supports nutrient absorption, immunity, and disease resilience.

**Results:**

We applied integrative multi-omics, combining untargeted LC-MS metabolomics and shotgun metagenomics, to explore the caecal responses of commercial Ross-308 broilers to two widely used gut health interventions: ionophore supplementation (T1) and anticoccidial vaccination (T2). Across 7,554 detected metabolites, we identified candidate metabolic signatures: T1 was marked by trends in prenol lipids, including multiple soyasaponins, and enrichment of cellular stress-related pathways (e.g. glutathione pathway). T2 instead was associated with shifts in aromatic amino acid metabolism, elevating tryptophan-derived indoles such as 5-methoxyindole. While global metabolic profiles did not differ significantly (PERMANOVA *p* > 0.05), supervised integration (DIABLO algorithm) identified 405 potential metabolite-MAG correlations. *Bacteroides fragilis* emerged as a dominant associate, correlating positively with a diverse range of metabolites (*n* = 271). Functional gene analysis suggested a link between *Mediterraneibacter* spp. and soyasaponin deglycosylation, while *Ruminococcaceae* UBA3818 showed genomic potential for tryptophan utilisation and indole-linked metabolic steps.

**Conclusion:**

Our exploratory findings suggest that prophylactic interventions impact the gut microbiome, resulting in divergent subsets of metabolic features. This highlights the potential of microbiome-informed strategies to improve enteric disease management and advance gut health centred approaches in both veterinary and human contexts.

**Supplementary Information:**

The online version contains supplementary material available at https://doi.org/10.1186/s42523-026-00596-z.

## Introduction

Gut health is a fundamental aspect of poultry production, directly influencing nutrient absorption, immune function, and overall performance [1]. Modern broiler chickens have been selectively bred for rapid growth, requiring an efficient digestive system to meet their high metabolic demands [2]. A well-balanced gut microbiota is central to this efficiency, as it facilitates digestion, protects against pathogens, and modulates immune responses [2]. However, disruptions to microbial communities, known as dysbiosis, can compromise gut integrity, reduce feed efficiency, and increase disease susceptibility, thereby affecting productivity and welfare in commercial poultry farming [1]. More widely, dysbiosis associated with enteric disease can increase water consumption and greenhouse gas emissions from poultry facilities [3, 4].

One of the most prevalent challenges to intestinal health is coccidiosis, a parasitic infection caused by protozoa of the genus *Eimeria* [5]. It is recognised as a significant economic burden, with a global cost exceeding £10.4 billion annually [5]. To mitigate its impact, the poultry industry primarily relies on ionophores— a class of coccidiostats that disrupt the ion balance in parasite cells [3, 6, 7]. Alternatively, live parasite vaccines can be used to induce immunity against specific species of the parasite, offering a proactive approach to disease management [8]. Ionophore use and vaccination are widely accepted strategies for coccidiosis control [9], but both have been shown to affect the gut microbiome in broilers significantly. For example, ionophores (monensin, salinomycin) have been reported to reduce microbial richness by depleting members of the *Ruminococcaceae* and *Clostridiales* groups [10]. Conversely, coccidiosis vaccination has been associated with decreased relative abundances of bacterial families like *Fusobacteriaceae*, *Gottschalkiaceae*, and *Lactobacillaceae* [11]. Although these studies demonstrate clear microbial shifts, the functional metabolic consequences of these changes remain poorly understood. This gap persists because most prior work has focused on microbiota composition alone, without determining how specific microbial taxa influence host-relevant metabolic pathways.

Advancements in metabolomics provide a promising avenue for exploring the functional outcomes of gut microbiome alterations [12]. Metabolomics involves the comprehensive analysis of small molecular metabolites (< 1500 Da) using high-resolution spectrometric methods [13]. Untargeted metabolomics, in particular, allows for the profiling of a wide array of metabolites without prior knowledge of their identity, offering a holistic view of the metabolic landscape [13]. When coupled with metagenomics, this integrated approach can uncover new biomarkers and functional relationships that cannot be detected through single-omics analyses [12]. Such approaches have begun to identify metabolites associated with *Eimeria* infection and vaccination status, including sphingolipids linked to vaccine responses [14].

Previously, we employed a metagenomic approach to assemble the genomes of gut microorganisms (as metagenome assembled genomes or MAGs) in broiler groups treated with different commercially available gut health interventions [15]. Comparison of ionophore and anticoccidial vaccination groups revealed notable differences in alpha diversity and the relative abundance of MAGs belonging to *Alistipes* spp., *Lactobacillus* spp., and *Cyanobacteria* [15]. Despite these insights into microbial ecology, studies integrating multi-omics data to establish links between microbiota composition, functional pathways, and host metabolism remain scarce [14, 16, 17]. This scarcity is partly due to limitations in applying in situ integration techniques, which have only matured in the past five years. Moreover, untargeted metabolomic analyses of caeca in broilers are, to our knowledge, currently lacking, limiting our understanding of how these widely used interventions alter the caecal metabolic environment.

In this work, we leverage recent advances in analytical strategies for integrative approaches [18] to address these gaps using broilers raised in a highly industrial setting. Specifically, we characterise the caecal metabolic landscape of commercial broilers exposed to ionophore or anticoccidial vaccine treatments and explore potential relationships between microbial communities and metabolite patterns. We hypothesised that ionophore treatment and anticoccidial vaccination would produce divergent caecal metabolic profiles that align with observed differences in microbial composition and functional gene pathways. Our integrated analysis is intended to highlight associations and generate testable hypotheses about microbe-metabolite interactions that may underlie these interventions. Our findings, therefore, provide preliminary insights into how gut health interventions shape the metabolic environment of the caeca of commercial broilers, offering a foundation for future mechanistic work and contributing to broader discussions of host-microbe interactions in veterinary and human health.

## Materials and methods

### Ethics statement

Poultry farm management and industry plant processing activities were conducted following Pilgrim’s Europe Ltd standard operating protocols, which are compliant with UK animal handling laws and regulations [19]. As part of the standard commercial practices of the company, all chickens were subjected to electrical stunning before slaughter and subsequent carcass processing [20].

### Study design and sample collection

Complete descriptions of the experimental design and general house management were detailed previously [15, 21]. In brief, a total of 6000 Ross-308 chickens were raised in an automated poultry house in Northern Ireland and provided a four-stage standard wheat-soy diet regimen [22] for 40 days. The birds were randomly allocated to 2 treatment groups with 6 replicate pens each (500 birds/pen): a control group (T1), where birds were provided ionophores (51 mg/kg) until day 30, and a vaccination group (T2), given a commercially available in-*ovo* vaccine against *Eimeria* species. On the last day of the production cycle (day 40), intact paired caecal samples were aseptically excised from the gastrointestinal tract within 5–10 min of the slaughtering process in a Pilgrim’s Europe Ltd. industry facility. Due to practical and financial constraints associated with untargeted metabolomics, we selected a subset of *n* = 10 independent biological replicates per treatment group from the same pens used for metagenomic sampling. Although driven by practical and financial constraints, a power analysis using the *pwr* package [23] (two-sided t-test, α = 0.05, power = 0.80) indicated that this sample size could detect only large effect sizes (Cohen’s d ≥ 1.3). Immediately following excision, caecal samples were then placed in sterile 50 ml tubes, maintained on ice, and immediately transported to the laboratory for cold storage at -80 °C until further use. In preparation for shipping, 500 mg caecal contents were aseptically collected from a randomly selected caecal pouch and transferred into 2 ml microcentrifuge tubes. Samples were then snap-frozen in liquid nitrogen for 15 min to preserve metabolic integrity.

### Metabolite extraction and untargeted metabolomics

Samples were sent in dry ice to BGI (Beijing, China) for untargeted metabolomics using Waters UPLC I-Class Plus (Waters, USA) paired with a Q Exactive High-resolution Mass Spectrometer (MS) (Thermo Fisher Scientific, USA). Chromatographic separation was carried out via Waters ACQUITY UPLC BEH C18 column (1.7 μm, 2.1 mm × 100 mm, Waters, USA) and Amide column (1.7 μm, 2.1 × 100 mm, Waters, USA) for nonpolar and polar metabolites, respectively. A full description of MS methods is available in the supplement information file.

### Metabolite ion peak extraction and metabolite identification

Mass spectrometry data was imported into Compound Discoverer 3.3 (Thermo Fisher Scientific, USA) and analysed using the BGI Metabolome Database, mzCloud™ (mzcloud.org), and ChemSpider™ (chemspider.com) [24] databases, from which a dataset containing basic chemical information including confidence level on matches, metabolite peak areas, and classification using the Human Metabolome Database (HMDB, hmdb.ca) [25] and the Kyoto Encyclopedia of Genes and Genomes (KEGG, genome.jp) [26] databases was obtained. Following the metabolite identification confidence workflow discussed previously [27], metabolites with identification reliability levels 1 to 4 (Level 1: Validated identification, Level 2: Putative identification, Level 3: Tentative structure – m/z database match, Level 4: Molecular formula) were retained in our dataset. For metabolites identified in both chromatographic methods, we selected identification results with a better identification confidence level and those with higher metabolite peak values. The final metabolomics dataset is available in the Data S1.

### Pre-processing and global overview of metabolomic data

Data was pre-processed through probabilistic quotient normalisation (PQN) normalisation using MetaX [28], quality control-based robust locally estimated scatterplot smoothing signal correction and removal of metabolites with high coefficient variation (> 30%) [29]. Metabolite counts were summarised at multiple data stages to track feature retention, while annotated metabolite relative abundances per HMDB superclass and KEGG superpathway hierarchies were distributed per sample. Proportions were normalised per sample to account for differences in total metabolite signal. Using the *vegan* R package, sample-wise variation between metabolite treatment groups was assessed using Principal Coordinates Analysis (PCoA) based on Euclidean distances, while differences in global metabolite composition were tested using permutational multivariate analysis of variance (PERMANOVA) at 999 permutations [30]. For all statistical analyses, a p-value of < 0.05 was considered significant.

### Functional analysis

Using *MetaboAnalystR* [31], we applied Mummichog, GSEA (Gene Set Enrichment Analysis), as well as their combined approach. Input data consisted of key metabolite information from both ion modes (positive and negative). We adopted a tolerance of 5 ppm while using the *Gallus gallus* gene set as background (cut-off threshold set at 0.05). Functional analyses were based on significant differences between the metabolite peak values of T1 and T2 that were assessed by student sample t-test and the Wilcoxon signed-rank test, for parametric and non-parametric data distributions, respectively, using base R [32]. KEGG pathways were further mapped using the R package *Pathview* [33], highlighting compounds enriched in either T1, T2, or in both. Some metabolites may exist in multiple chemical forms (chemical isomers and adducts), they were grouped under the same compound identification where appropriate.

### Multivariate analysis methods for biomarker identification

Potential biomarker identification was conducted using Least Absolute Shrinkage and Selection Operator (LASSO) regression, implemented through the *glmnet* package with 10-fold cross-validation [34]. LASSO applies an L1 penalty term, which forces the coefficients of less predictive variables to shrink to zero, effectively performing variable selection [34]. This process retains only those metabolites with significant predictive contributions. The metabolites with non-zero coefficients were then used to differentiate between the binary outcomes of T1 (“No”) and T2 (“Yes”), coded as 0 and 1.

We further applied the sparse partial least squares discriminant analysis (sPLS-DA) algorithm from the *mixOmics* package on our PQN-normalised data to perform feature selection, treating the treatment categorisation as a latent variable [35]. The sPLS-DA algorithm selects features by identifying a subset of variables that best discriminates between predefined classes, while simultaneously reducing data dimensionality. It introduces sparsity in the latent components by also applying L1 regularisation (similar to the LASSO approach, however, applied on coefficients of fitted principal components), which shrinks the coefficients of less important variables to zero, allowing only the most relevant features to contribute to the classification model [35]. Model performance was assessed via the *perf* function through an M-fold cross-validation strategy (CV) strategy (5-fold x 10 repeats). Discriminative power was quantified using the classification error rate (CER) (ranging from 0 to 1) and Area Under the Curve (AUC). To ensure the robustness of the identified biomarkers, we assessed the selection stability (defined as the frequency with which a variable was selected across all cross-validation folds) and importance (absolute value of its loading coefficients, which represents its individual contribution to the latent components) of each metabolite.

### Chemical enrichment analysis

Building on the significant biomarkers identified through sPLS-DA, we collected the key chemical identifications for each biomarker, including Simplified Molecular Input Line Entry System (SMILES), PubChem ID, and International Chemical Identifier (InChIKey) data, from the PubChem [36] and ChemSpider [24] databases. This information was subjected to chemical class enrichment using basic ChemRICH analysis [37] (available at https://chemrich.idsl.me/). This method utilises an algorithm that categorises selected metabolites based on their Medical Subject Headings (MeSH) classifications, leveraging chemical similarity and ontologies. Groups with similar classes are then considered as a set for the Kolmogorov-Smirnov test to obtain the set-level significance [37]. We also employed ChemRICH class enrichment analysis of the same features, but with classification based on the ChemOnt system via the ClassyFire program [38].

### Integration analysis of metagenome and metabolome data

We applied the Data Integration Analysis for Biomarker discovery using Latent cOmponents (DIABLO) method from the *mixOmics* package [35, 39] to determine the correlation between our metabolome and MAG datasets. A total of 84 MAGs with > 75% completeness and < 5% contamination were previously obtained [15]. Before integration, MAG abundance was normalised using Total Sum Scaling and Centered Log Ratio (TSS + CLR) method as we performed previously [15]. To prevent overfitting an ensure an optimal feature-to-sample ratio, we performed automated parameter tuning using the *tune.block.splsda* function. This step identified the optimal number of variables to retain per component by minimising the cross-validated Balanced Error Rate (BER).

The final DIABLO model was built to maximise separation between the treatment groups while considering correlations between the datasets. A design matrix with a link set at 0.10 (based on the recommendation of the original authors of mixOmics, this value represents a reasonable trade-off between discrimination, i.e., T1 and T2, and correlations across metagenomics and metabolomic datasets) was used to discriminate features between the treatment groups. Classification performance and the global BER were evaluated using 5-fold cross-validation and repeated 10 times using the *perf* function. Feature selection was further validated by calculating frequency stability metrics across all cross-validation folds to identify the most robust discriminatory markers. The statistical significance of the integrated model was then confirmed using AUC values and a permutation test (via the *auroc* function), which compared the performance of the original model against a null distribution generated from randomly permuted group labels (*p* < 0.05).

From the model, moderate correlations between MAGs and metabolites of at least 0.60 were retained and thereafter subjected to pairwise comparison with Benjamini-Hochberg p-value correction (*p* < 0.05). Both significant and non-significant correlation counts, CV stability values, and selected metabolic module features of significant MAGs were mapped together using the *ComplexHeatMap* package for complete clustering analysis based on the Euclidean method [40]. Data on the metabolic modules were collated from our previous study [15] and then classified at the KEGG module class level [26]. Correlations between significant MAGs and metabolites based on DIABLO, with further evidence based on sPLS-DA, were also drawn using the *igraph* package [41]. Other parameters used in the sPLS-DA and DIABLO algorithm workflows are provided in the supplementary file.

To further build on significant DIABLO metabolite-MAG relationships, we further processed genomes of selected MAGs using prodigal 2.6.3 [42] for protein prediction; generated protein sequences were then annotated for KEGG orthology (KO) genes using KofamKOALA ver. 2025-05-01, using default settings [43]. Pathways facilitating metabolism of significant pathways were also mapped using the KEGG mapper tool [44] and related literature available on soyasaponin [45, 46] and indole metabolism [47].

## Results

### Global metabolome structure and composition of caecal samples across treatment groups

**Fig. 1:**
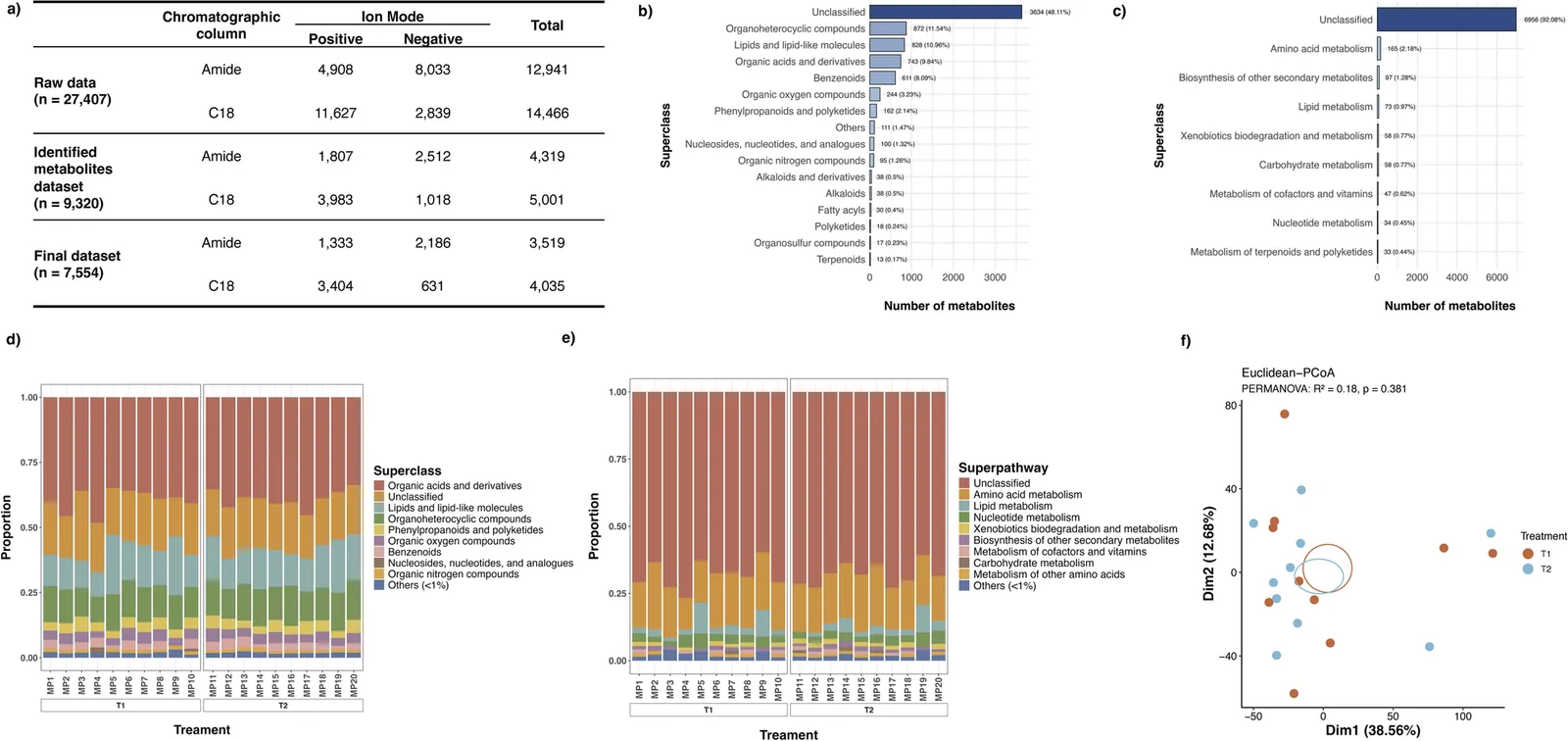
Global overview of metabolite distribution across treatment groups. (**a**) Summary of metabolite counts across processing stages. Metabolite counts grouped according to (**b**) HMDB superclass level and (**c**) KEGG Superpathway level. Proportional distribution across individual samples within each treatment group (T1 and T2, *N* = 20) at (**d**) HMDB superclass level and (**e**) KEGG super pathway level. (**f**) Principal coordinates analysis (PCoA) based on Euclidean distances with PERMANOVA statistics indicating no significant separation between groups (R² = 0.18, *p* > 0.05)

A total of 7,554 putative metabolites were detected across all caecal samples, with 3,519 identified from the amide column and 4,035 from the C18 column (Fig. 1a). The metabolites were categorised by identification confidence: 172 (2.2%) were identified at level 1, 1206 (16%) at level 2, 1599 (21.2%) at level 3, and the remaining 4577 (60.6%) were at level 4.

Metabolite annotation also revealed a substantial proportion of unclassified compounds, comprising 48.11% at the HMDB superclass level and 92.08% at the KEGG pathway level (Figs. 1b–c). Among the annotated HMDB superclasses, organic heterocyclic compounds (11.54%) and lipid and lipid-like molecules (10.96%) were the most prevalent (Fig. 1b). In contrast, amino acid metabolism pathways (2.18%) dominated the classified KEGG superpathway annotations (Fig. 1c). Metabolite proportion levels for both classifications remained largely consistent, showing minimal variation across T1 and T2 samples (Figs. 1d–e). Furthermore, beta diversity analysis using PCoA based on Euclidean distances and PERMANOVA revealed no statistically significant separation between treatment groups (R² = 0.18, *p* > 0.05; Fig. 1f). Full chemical information for all metabolites is listed in Data S1.

### Administration of treatments significantly impacts metabolites involved in various amino acid metabolism pathways

**Fig. 2:**
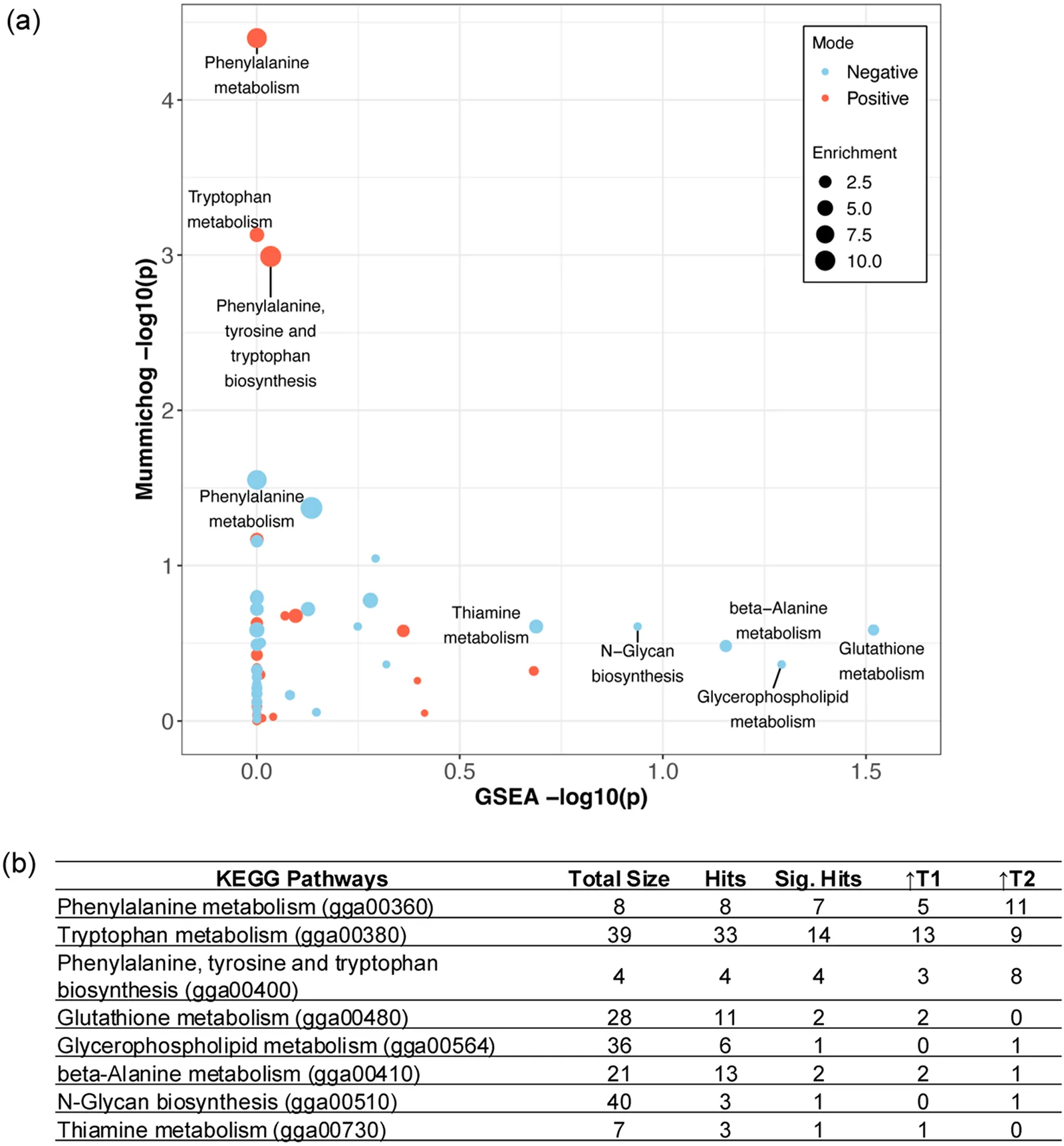
Functional analysis of all 7554 metabolites. (**a**) Enrichment plot of functional pathways according to Mummichog and GSEA, showing *p* values of top pathways and enrichment factor (ratio of number of significant hits and total size) of top enriched pathways. (**b**) List of top enriched pathways and corresponding KEGG compound frequency in T1 and T2

Pathway enrichment analysis between T1 and T2 was performed using Mummichog and GSEA. Based on mummichog analysis, significantly enriched pathways (*p* < 0.05) include *Phenylalanine metabolism (Phe)*, *Tryptophan metabolism (Trp)*, and *Phenylalanine*,* tyrosine and tryptophan biosynthesis (Phe-Tyr-Trp)* (Fig. 2). Meanwhile, according to GSEA analysis, only *glutathione metabolism* was significantly altered (*p* < 0.05). Nevertheless, enrichment was also notable for pathways: *Glycerophospholipid metabolism* (*p* = 0.051), *beta-Alanine metabolism* (*p* = 0.07), *N-glycan biosynthesis* (*p* = 0.12) and *thiamine metabolism* (*p* = 0.21). Complete enrichment results are shown in Data S1.

**Fig. 3:**
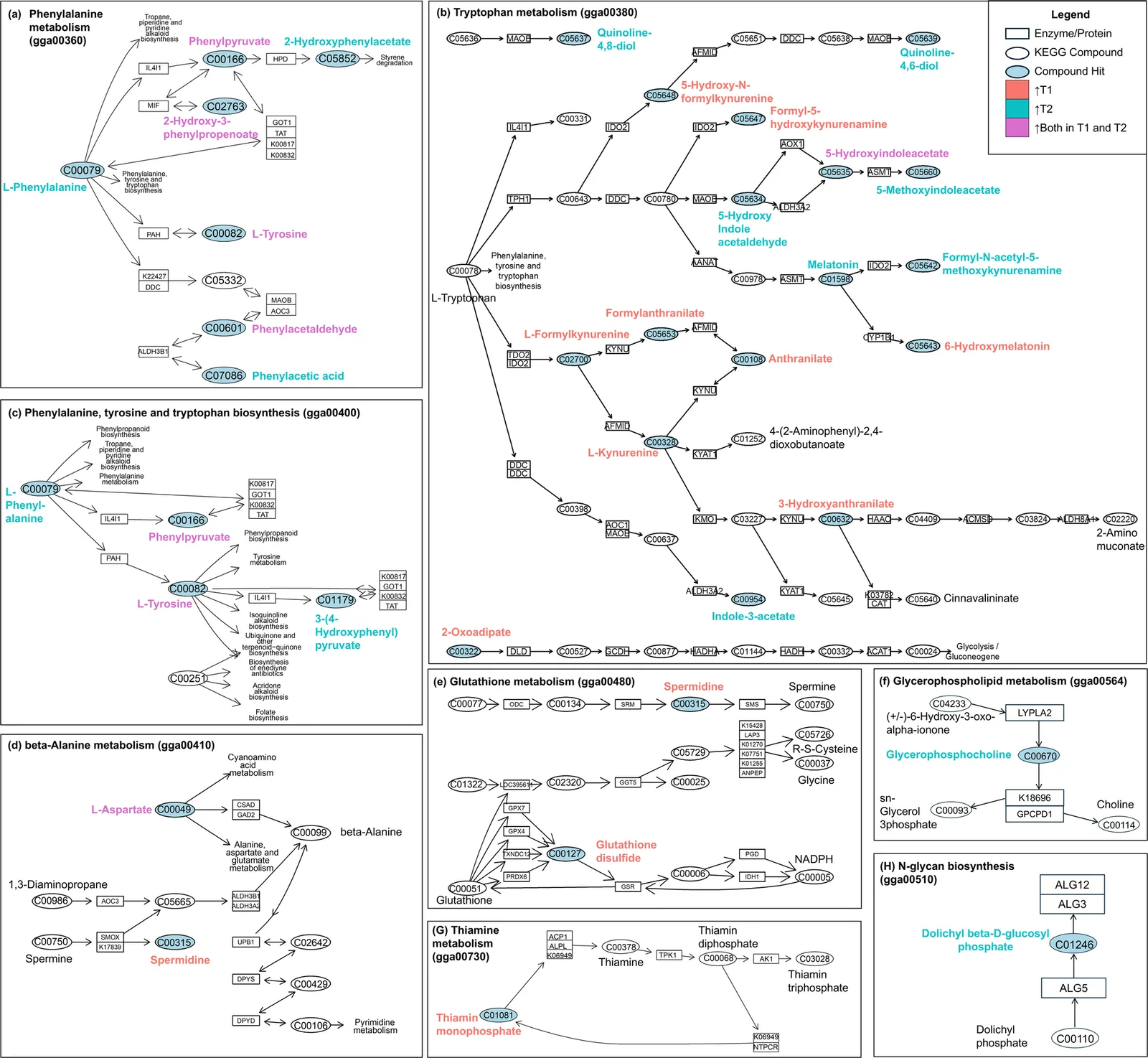
Pathway mapping of KEGG metabolites included in enriched KEGG pathways. Compound names in red and aqua green indicate higher numbers of significant hits in T1 and T2, respectively; Names in purple indicate the same number of significant hits

We also mapped the top enriched pathways, highlighting compounds that are enriched in either T1 or T2 (Fig. 3). The number of enriched compounds in metabolism pathways Phe, Phe-Tyr-Trp, *glycerophospholipid* and *N-glycan biosynthesis* were observed as significantly higher in T2, while the enriched compounds in *beta-Alanine*, *glutathione* and *thiamine* metabolism pathways were higher in T1. For the *Trp pathway*, 9 of the 16 unique compounds, many of which are formylkynurenine-based metabolites, were greater in abundance in T1, while the remaining 7 compounds, which are later stages of the pathway, such as melatonin and quinoline diols, were elevated in T2.

### Discriminant analysis of metabolites identified robust biomarkers primarily from prenol lipids and indoles

Since there is a lack of global separation in the caecal metabolome as stated previously (Fig. 1f), we employed two supervised discriminant analyses (LASSO regression, sPLS-DA) to isolate specific high-variance discriminatory markers from the high-noise background of the global metabolite profile. The LASSO analysis (λ = 0.0039) revealed 12 differentially abundant metabolites, evenly distributed across the two treatment groups (Table 1), wherein metabolites with negative coefficients represent T1, while those metabolites with positive coefficients represent T2. Compound C4316 (iPrPh-GlcA) had the lowest coefficient, followed by C447 (NP-018545) and A1176 (Soyasaponin III), suggesting a stronger association with T1. In contrast, C1445 had the highest coefficient, indicating the strongest correlation with T2, which is followed by C1548 and C1983.

**Table 1:** LASSO regression* coefficients of differential metabolites in T1 (control) and T2 (vaccination) groups

ID	Name	RT	m/z	Formula	Level	Superclass	Class	Coefficient
-	-	-	-	-	-	-	*Intercept*	-1.646
C4316	(2 S,3 S,4 S,5R)-3,4,5-Trihydroxy-6-(5-methyl-2-propan-2-ylphenoxy)oxane-2-carboxylic acid	4.693	327.14508	C_16_H_22_O_7_	4	Lipids and lipid-like molecules	Prenol lipids	-2.892 × 10^− 6^
C447	NP-018545 (2-Hydroxy-4-methoxy-7-methyl-7,8,9,10,11,12,13,14-octahydro-6-oxabenzocyclododecen-5-one)	7.005	291.1597	C_17_H_24_O_4_	2	Phenylpropanoids and polyketides	Macrolides and analogues	-1.837 × 10^− 6^
A1176	Soyasaponin III	2.431	795.45752	C_42_H_68_O_14_	3	Lipids and lipid-like molecules	C30 isoprenoids	-5.881 × 10^− 7^
A3078	3-Hydroxyisovalerylcarnitine	1.701	262.16497	C_12_H_23_NO_5_	4	Lipids and lipid-like molecules	Fatty Acyls	-1.756 × 10^− 7^
A4058	Penem	3.197	126.00219	C_5_H_5_NOS	4	Organoheterocyclic compounds	Lactams	-1.618 × 10^− 7^
A508	Phenylethanolamine	1.488	138.09161	C_8_H_11_NO	2	Organic nitrogen compounds	Organonitrogen compounds	-3.639 × 10^− 8^
C3313	Fluvoxamino acid	5.669	319.12799	C_14_H_17_F_3_N_2_O_3_	4	Benzenoids	Benzene and substituted derivatives	5.7622 × 10^− 8^
C861	5-Methoxyindole	5.509	148.07593	C_9_H_9_NO	2	Organoheterocyclic compounds	Indoles and derivatives	1.0847 × 10^− 7^
C218	NP-015145 (Flindersine)	3.503	250.08276	C_14_H_13_NO_2_	2	Organoheterocyclic compounds	Quinolines and derivatives	1.3381 × 10^− 7^
C1983	18-HETE	9.53	299.25803	C_20_H_32_O_3_	3	Lipids and lipid-like molecules	Eicosanoids	1.6213 × 10^− 7^
C1548	N1-(2,3-dihydro-1,4-benzodioxin-2-ylmethyl)-2,2-dimethylpropanamide	6.625	250.1439	C_14_H_19_NO_3_	3	Organoheterocyclic compounds	Unclassified	1.6529 × 10^− 7^
C1445	3-methyl-2,4-dioxo-1-phenyl-1,2,3,4-tetrahydropyrimidine-5-carbonitrile	5.236	266.03225	C_12_H_9_N_3_O_2_	3	Unclassified	Unclassified	1.6759 × 10^− 6^

*Least absolute shrinkage and selection operator (LASSO) regression against metabolites where the treatment groups are coded as 0/1, corresponding to T1/T2. The model assumes an L1 penalty term, which forces the beta coefficient of insignificant predictors to become zero. RT: Liquid chromatography retention time, m/z: mass-to-charge ratio

**Fig. 4:**
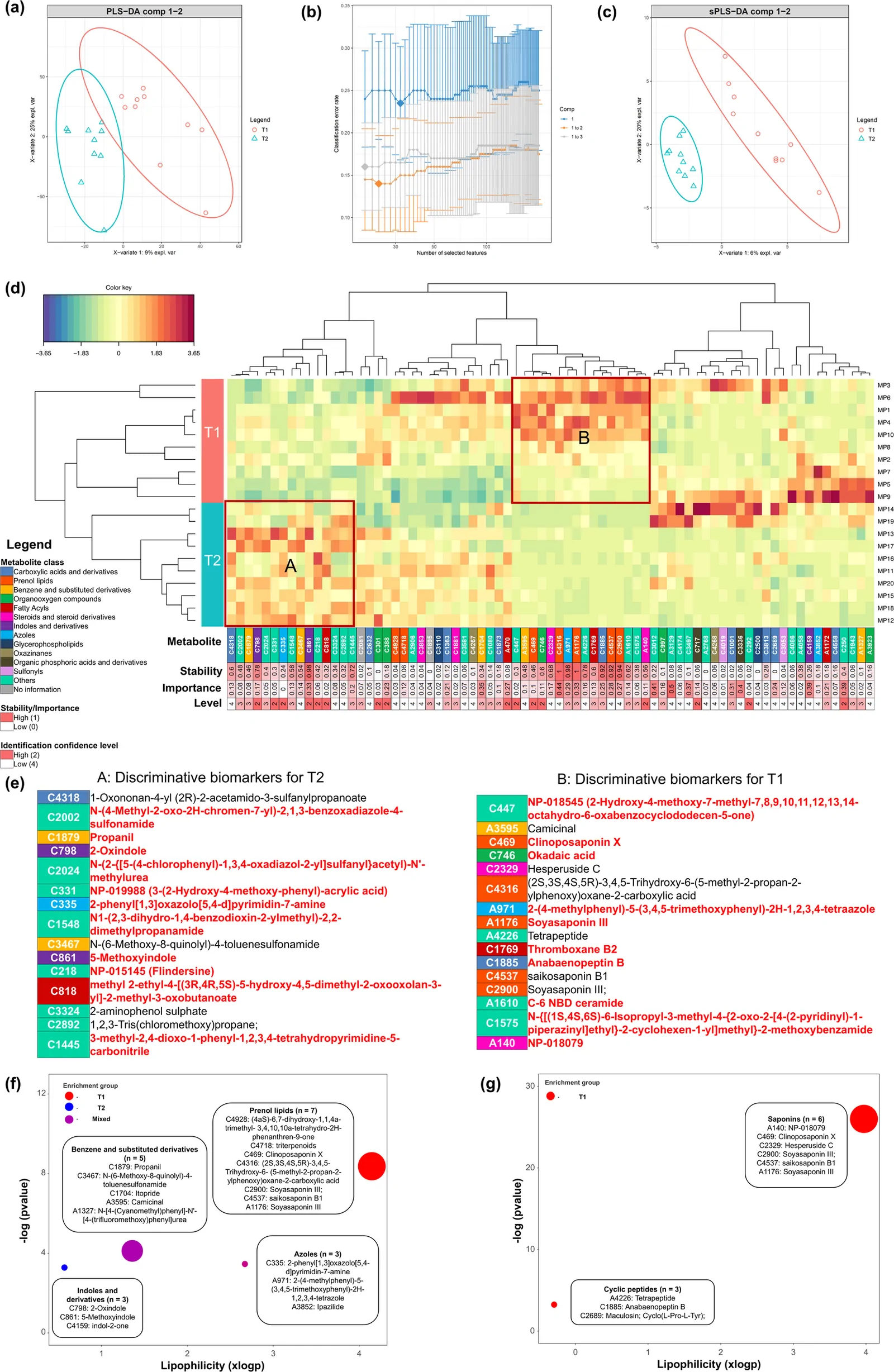
Biomarker identification using sPLS-DA and chemical enrichment between treatment groups. Panel (**a**) shows samples spanned by the two main components derived from partial least squares discriminant analysis (PLS-DA). Panel (**b**) exhibits the classification error rate of each component across the total number of selected features. Panel (**c**) shows samples spanned by the two main components derived from spares PLS-DA (sPLS-DA). (**d**) Heatmap illustrating the classification of samples based on the most discriminating metabolites derived from sPLS-DA. The left dendrogram branches into three major nodes, two nmodes representing T1 and one nodethe other representing T2 samples. The top dendrogram branches into 6 minor nodes, of which 2 nodes – A (15 metabolites) and B (16 metabolites) contain the most robust features (having high values for both importance and stability) for T2 and T1, respectively. (**e**) List of representative metabolites for T1 and T2. Metabolites with identification classification levels 1–3 are indicated in red. Chemical enrichment analysis was based on classifications according to the (**f**) ChemOnt system and (**g**) MeSH system. A minimum of 3 metabolites per cluster was considered, and set-level significance was set at *p* < 0.05. Key message: Multivariate modelling (sPLS-DA) identified a subset of discriminative metabolites that separate the treatment groups with enrichment analyses suggesting prenol lipids and cyclic peptides to represent T1 and indoles and derivatives to represent T2

The sPLS-DA analysis was conducted in several steps to identify discriminative metabolites between the two treatment groups. Initially, a PLS-DA was performed, where the first two components accounted for 9% and 25% of the variance, respectively (Fig. 4a). Following model tuning, the sPLS-DA selected 32 features for component 1 (Comp1), 24 for component 2 (Comp2), and 20 for component 3 (Comp3) (Fig. 4b). Metabolite A4058 was selected both in Comp1 and Comp2, yielding a selection total of 75 unique features (Data S1). Comp1 of the sPLS-DA model explained 6% of the variance, while Comp2 accounted for 20% (Fig. 4c). Model performance was evaluated, yielding an average classification error rate of 21% (Supplement Fig. 1), with AUC values: 0.84 for Comp1 and 0.87 for Comp2.

The heatmap consisting of 75 metabolites generated from sPLS-DA (Fig. 4d) demonstrated clear separation between the T1 and T2 samples. Of these, 16 (21.3%) were identified at reliability level 2 and 23 (30.7%) at level 3. The remaining 36 features (48%) were classified as level 4. Subclusters A and B include selected metabolites with stability (ranges from 0.3 to 1) and importance (ranges from 0.02 to 0.44), representing T2 (*n* = 15 features) and T1 (*n* = 16 features), respectively. Highlighted in red are also those reliably identified (at most level 3) (Fig. 4e). Within subcluster A, C861 (5-methoxyindole) showed the highest stability and importance, followed by C798 (2-oxindole), both of which were categorised as level 2 metabolites. Meanwhile, notable discriminatory subcluster B metabolites with reliable levels include A1176 (Soyasaponin III) and A971 (a tetraazole). Based on ChemOnt classification, the metabolites in cluster A are predominantly prenol lipids (PL), while cluster B features metabolites such as 5-methoxyindole and 2-oxindole, which belong to the indoles and derivatives (IND) group.

For confirmation of enrichment by chemical class in the treatment groups, we further investigated the 75 metabolites selected by sPLS-DA analysis by applying ChemRICH class enrichment analysis, in which we used two classification systems –ChemOnt and MeSH. A detailed list of metabolite classification for each system is available in Data S1. ChemOnt-based enrichment revealed 4 clusters, corresponding to groups IND, azoles, benzenes and derivatives, and PL (Fig. 4f). The PL group, which was composed of 7 metabolites, was enriched in T1, while a cluster of 3 metabolites belonging to IND was enriched in T2. The remaining clusters were neither enriched in T1 nor T2. Based on the MesH system, only two class clusters are significant, namely saponins (5 metabolites) and cyclic peptides (2 metabolites), both of which are enriched in T1. Interestingly, 5 metabolites belonging to the saponin cluster (A1176: Soyasaponin, C2900: Soyasaponin III, C469: Clinoposaponin *X*, C4537: saikosaponin B), belong as well to the PL group (Fig. 4g).

### Integrative omics highlights microbial contributions to caecal metabolic variation

**Fig. 5:**
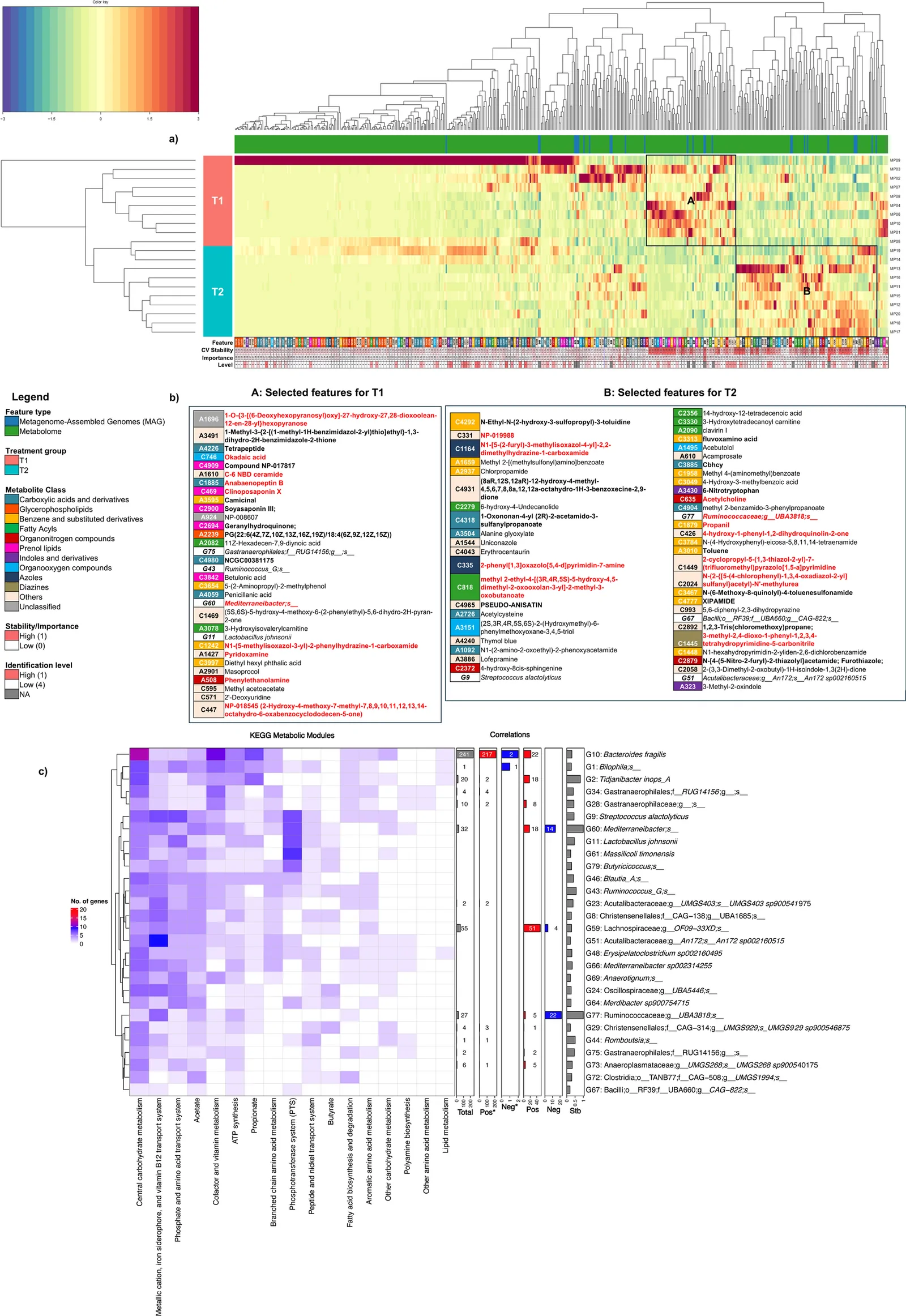
Integrated discriminative analysis of metagenomic and metabolic features between T1 and T2 caecal samples based on the DIABLO algorithm. (**a**) Heatmap illustrating the classification of samples based on the most discriminating features. The left dendrogram branches into 3 major nodes, exhibiting clear separation of T1 (first 2 nodes) and T2 (third node) samples. The top dendrogram branches into four major nodes, one of which we marked as cluster A, containing 32 features and another as cluster B, covering 51 features. Clusters A and B contain the most robust features (those with high values for both importance and stability) for T1 and T2, respectively. (**b**) List of discriminative features for T1 and T2. Metabolite names in red indicate reliability identification levels 1 - 3; Names of metabolites with >0.70 stability indicated in bold. (**c**) Heatmap showing significant MAGs from DIABLO analysis and their KEGG module capacities (selected in relation to enriched metabolic pathways in Fig. 2). The number of significant and non-significant metabolite correlations and the stability of each MAG are also shown. Key message: DIABLO integration reveals multi-omics signatures for each treatment group, highlighting correlated MAGs and their associated functional potential

Our DIABLO model revealed moderate to high correlation between the metagenome and metabolome features across the three major components (Comp1: 62%, Comp2: 79% and Comp3: 85%). The optimal number of variables consisted of a total of 188 unique features – 28 MAGs (10 in each component) out of 84 MAGs and 160 metabolites (55 in Comp1, 90 in Comp2 and 16 in Comp3) out of 7554 metabolites (some of the variables were selected in more than 1 component). A detailed list of selected features and stability scores is shown in Data S1. Based on performance permutation testing and AUC calculation, the model demonstrated high discriminatory performance, achieving an AUC of 1.0 (*p* < 0.001) for the metabolome block and an AUC > 0.96 (*p* < 0.001) for the metagenome block across all components (Fig. 5).

Group separation of samples by abundance was accomplished by DIABLO, as shown in Fig. 5, which covers 237 selected features (12 MAGs, 225 metabolites). Two major branches (A and B), highlighted due to the high values for feature stability and importance, serve as reliable markers that discriminate between the two treatments. Node A, which includes 32 features (4 MAGs, 28 metabolites), best represents T1, while T2 is best represented by node B, which includes 51 features (4 MAGs, 46 metabolites) (Fig. 5b). A small proportion (19/237, 8%) of these selected metabolites were similarly selected as by sPLS-DA analysis, such as C746 (Okadaic acid) and C2900 (Soyasaponin III;).

A total of 405 unique correlations between metabolites and MAGs were identified using the DIABLO model, with more than half (235/405) being statistically significant (*p* < 0.05) based on pairwise comparisons (Data S1). Among the selected MAGs, G10: *Bacteroides fragilis* had the highest number of correlations (*n* = 241), with the majority being positive and significant (217/241) (Fig. 5c). Other MAGs with a high number of positive correlations included G2: *Tidjanibacter inops A*, G60: *Mediterraneibacter*, and G59: Lachnospiraceae *OF09-33XD*. In contrast, G77: Ruminococcaceae *UBA3818* exhibited the highest number of negative correlations (*n* = 22). Additionally, MAGs G2, G59, and G77 showed the greatest stability based on cross-validation assessments.

Due to the significant relationship of these MAGs with the metabolome, we also explored corresponding microbial metabolic KEGG modules that may be attributed to their correlation with the metabolites. We included modules primarily on energy, amino acid, and vitamin metabolism to build on the results of enrichment analysis (Fig. 2). Heatmap clustering of these modules resulted in 5 subclusters, representing different levels of metabolic capacities. Interestingly, G10, which has both the greatest number of correlations and modules, is in the topmost cluster, along with MAGs G1 and G2. Meanwhile, the middle cluster, composed of 6 MAGs including G9 and G59, has a notably high number of PTS modules, while the last cluster consists of 7 MAGs including G77.

**Fig. 6:**
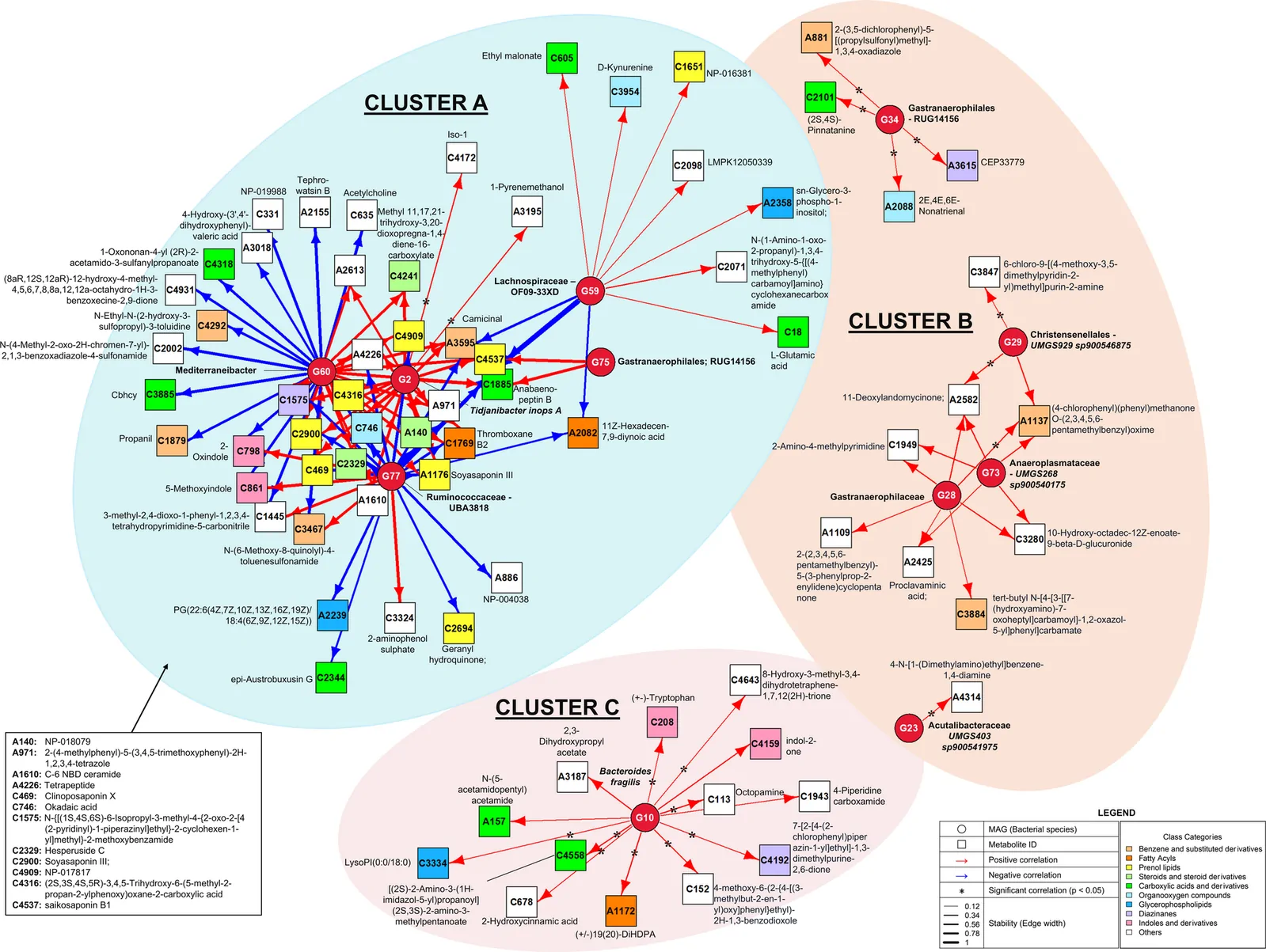
Network showing selected correlations between MAGs and metabolites identified through DIABLO analysis. Correlated metabolites are classified according to the ChemOnt system. The network was subdivided into clusters A, B and C based on the proximity of related nodes. *Significant correlations are based on pairwise comparison with Benjamini-Hochberg correction (*p* < 0.05)

Notable correlations were mapped in a network plot that is composed of 3 major clusters (A, B and C) (Fig. 6). Correlations shown in cluster A predominantly involve discriminatory metabolites derived from our sPLS-DA model, where we observe that G77 generally has a negative relationship with T1 markers such as PLs (C4316, A1176, A3595, C1575, C469, C746, A140, C2900) and a positive relationship with T2 IND markers (C798, C861). In contrast, G2 and G60 are observed to have a positive correlation with the same T1 marker metabolites, while G60 has a negative impact on T2 IND markers along with other 10 metabolites such as A2155, C4931, and C1445. Meanwhile, G59 has a couple of positive correlations with carboxylic acids and derivatives (C605: Ethyl malonate and C18: L-Glutamic acid) and with C3954: D-Kynurenine. Clusters B and C include positive correlations between MAGs (G34, G29, G73, G28, G23, and G10) and metabolites belonging to a variety of chemical classes. Notably, G10 is shown to be highly correlated (> 0.85) with multiple amino acids, including C208: (+/-) Tryptophan (Fig. 6c). Amino acids, peptides and analogues are also the predominant subclass among metabolites correlated to G10 (Data S1).

### Microbial genomic potential associated with soyasaponin and tryptophan-linked metabolic patterns

**Fig. 7:**
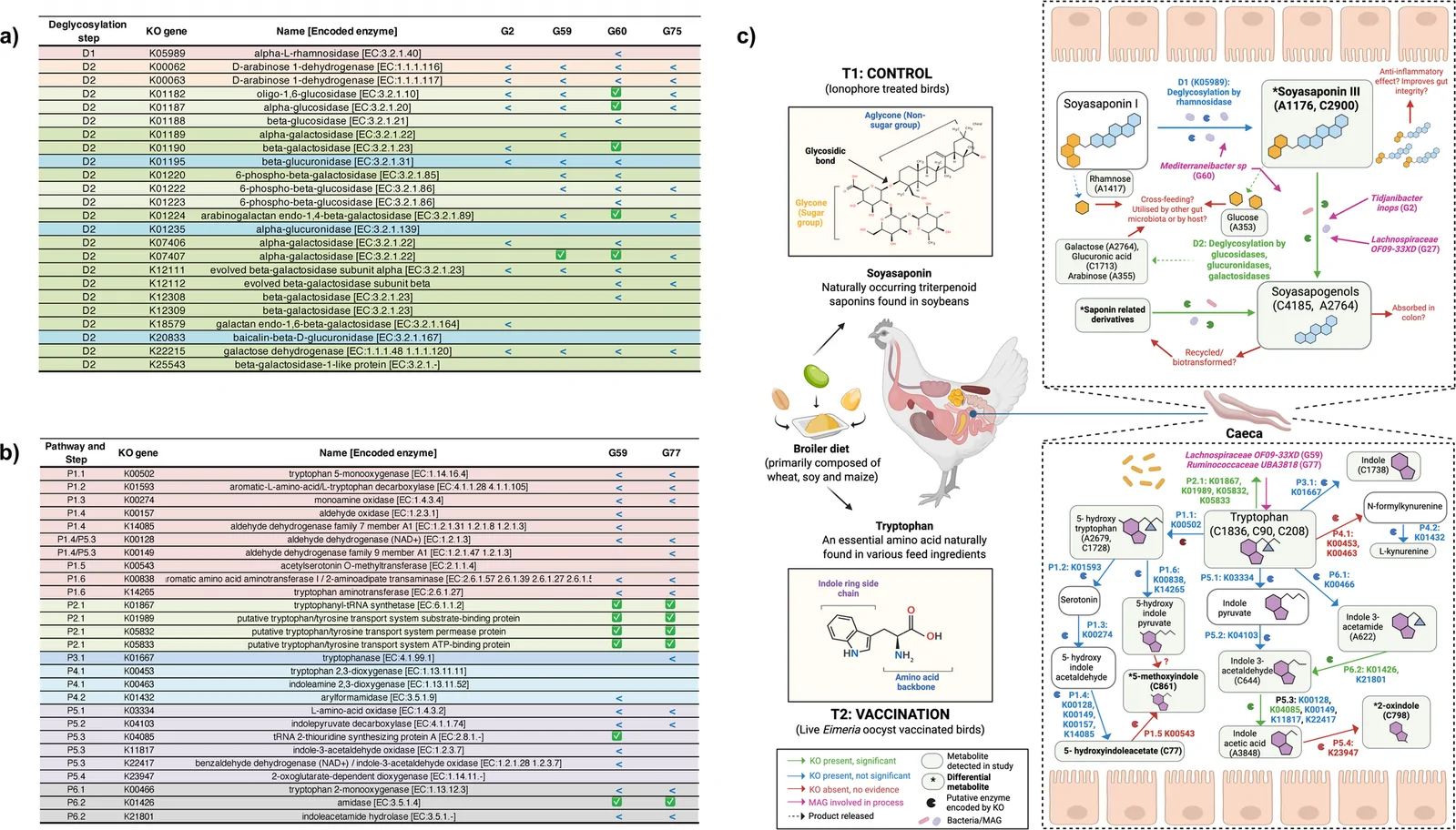
Putative soyasaponin and indole metabolism pathways as influenced by prophylactic methods. Detected KO genes and corresponding information in (**a**) MAGs correlated to soyasaponin degradation and (**b**) MAGs correlated to indole derivatives. (**c**) Mapping of genes putatively involved in the metabolism of differential compounds detected in the caeca. KO: KEGG orthology; EC: Enzyme commission number. G2: *Tidjanibacter inops*; G59: Lachnospiraceae OF09-33XD; G60: *Mediterraneibacter sp*.; G75: Gastranaerophilales; f__RUG14156; G77: Ruminococcaceae *UBA3818*

To elucidate the role of key MAGs in driving distinct metabolic outcomes across groups, we annotated KOs from the metagenomes of G2, G27, G60, G75, and G7 using Prodigal for gene prediction and KofamKOALA for KO assignment. The KO profiles for each MAG are provided in Data S2. These profiles were subsequently screened for corresponding enzymes involved in soyasaponin and tryptophan metabolism (Data S2 and Fig. 7).

In ionophore-treated birds (Fig. 7a and c), a stepwise microbial transformation of soyasaponin I occur within the gut environment is proposed, primarily involving deglycosylation reactions. A conversion of soyasaponin I to soyasaponin III (A1176, C2900) is mediated by microbial rhamnosidase activity (K05989), with G60 identified as a putative microbial agent facilitating this step. Soyasaponin III can be further metabolised by glycosidases such as glucosidase, glucuronidase, and galactosidase (Step D2, Fig. 7c) yielding soyasapogenols that we also detected in our dataset, including Juvenate (C4185) and 7C-Aglycone (A2764). Breakdown of other soyasaponins is also putatively facilitated by MAGs G60, G2 and G27, which were shown to have differing capacities for deglycosylation, indicating probable release of various sugar moieties, including sugar compounds that we detected in our dataset: glucose (A353), galactose (A2676), glucuronic acid (C17113), and arabinose (A355).

Meanwhile, in the caeca of vaccinated birds, we were able to map 6 possible pathways (P1 - P6) for tryptophan metabolism, leading to the production of multiple bioactive indole-related metabolites (Fig. 7b and c). Alongside tryptophan itself, we detected 8 tryptophan derivatives in our dataset that are precursors to the differential T2 metabolites: 5-methoxyindole and 2-oxindole. While the MAGs G59 and G77 do not possess a complete set of genes involved in tryptophan degradation, our data shows genetic evidence of statistically significant transport and utilisation of tryptophan (P2.1 genes) and 2 KO genes that encode for enzymes (P5.3, P6.2) that catalyse the breakdown of precursor molecules into 2-oxindole. Both G59 and 677 were both observed for potential capacity towards breakdown of tryptophan via the P1 pathway, while G59 was observed to have a higher potential for tryptophan breakdown via the P5 pathway.

## Discussion

A stable and balanced interaction between the gut microbiota and its host is critical for maintaining overall health and ensuring proper physiological functions in poultry. However, infectious challenges such as coccidiosis can severely disrupt this equilibrium, leading to microbial imbalances and impairing the functional contributions of the gut microbiota to the host [48]. The primary strategies for controlling coccidiosis in chickens are dietary supplementation with ionophores or vaccination using varied formulations of live *Eimeria* parasites, both of which are highly effective and reliable [9]. Nevertheless, as the poultry industry faces evolving challenges, refining these conventional methods requires a deeper understanding of their mechanisms in modulating gut health. In previous work, we assessed the effects of these control strategies on the caecal metagenome, revealing significant changes in alpha diversity, the abundance of specific microorganisms, and carbohydrate digestion functions [15]. However, the specific metabolites associated with these microbial alterations remained uncharacterised, leaving open the question of how such shifts translate into functional effects. The gut microbiota, often regarded as a vital endocrine organ, produces numerous bioactive molecules. These metabolites influence not only the local gut environment but also systemic processes, triggering physiological responses that shape host health and behaviour [49]. To further understand the impact of coccidiosis control strategies, we employed high-throughput LC/MS untargeted metabolomics in combination with metagenomics. With this integration, we moved beyond descriptive microbial profiling to a functional systems-level understanding of how gut microbiota and host metabolism interact in response to interventions [18]. This approach enabled preliminary identification of key metabolic pathways and bioactive compounds that mediate the effects of these strategies.

The chicken caeca are central to broiler digestive efficiency, serving as the primary site for microbial fermentation of undigested nutrients, which produces short-chain fatty acids (SCFAs) that supply additional energy to the host [50]. They also absorb water, electrolytes, and nitrogenous compounds like ammonia, which can be recycled into microbial protein [50]. While metabolomic studies on caeca remain limited, an NMR-based study confirmed that they contain high levels of SCFAs, amino acids, and notably O-phosphocholine, a metabolite associated with immune responses against bacterial infections [51, 52]. In our study, a total of 7,554 metabolites were detected throughout all samples, with a considerable portion of these metabolites unclassified under existing metabolic databases such as HMDB and KEGG.

High proportion of unclassified compounds are common in untargeted metabolomics, particularly in non-model organisms such as poultry [53]. This largely reflects the limited coverage of current metabolite reference databases, which are biased toward human, plant, and well-characterised microbial metabolites. Many gut-derived compounds, especially those produced by commensal bacteria, anaerobic fermentations, or complex diet-microbiota interactions, lack reference MS/MS spectra or structural annotations in publicly available libraries. As a result, a substantial fraction of features in caecal metabolomes cannot be assigned to known chemical classes or pathway identifiers [54]. This also highlights the need for further metabolomic research, especially given the microbiome’s growing recognised role in poultry metabolism and productivity [55, 56]. Nonetheless, among identified metabolites, our analyses revealed organic heterocyclic compounds and lipid-like molecules as the most abundant HMDB metabolite classes. The predominance of organic heterocyclic compounds aligns with their essential roles in diverse biological functions and their adaptability for various medicinal applications [57]. Meanwhile, lipid and lipid-like metabolites have been highlighted in previous poultry metabolomics research due to their roles in energy homeostasis and cellular signalling [17, 58].

An initial finding in our study is the lack of global metabolic divergence between treatment groups, as indicated by our PCoA and PERMANOVA results; However, further pathway enrichment and supervised analysis suggested that the impact is targeted toward specific pathway shifts rather than inducing a broad, systemic reorganisation of the caecal metabolome. Pathway enrichment analysis revealed distinct metabolic adaptations between treatment groups: T1 (ionophore-treated) birds showed significant enrichment in the glutathione metabolism pathway, with elevated levels of spermidine and glutathione disulfide. Spermidine, a polyamine derived from putrescine, is known to enhance CD8 + T cell metabolism via fatty acid oxidation [59]. However, the concurrent increase in glutathione disulfide, the oxidised form of glutathione, may indicate elevated oxidative stress [60]. Additionally, increased thiamine monophosphate in T1 suggests enhanced thiamine turnover, potentially linked to Eimeria metabolism or the action of amprolium, a thiamine analogue used in coccidiosis control [61, 62]. T2 (vaccinated) birds exhibited enrichment in aromatic amino acid metabolism, particularly phenylalanine, tyrosine, and tryptophan. These essential amino acids, which must be obtained from diet or microbial synthesis, are precursors for neurotransmitters like serotonin and dopamine [63]. Their enrichment suggests increased microbial activity in neurotransmitter precursor production [64]. T2 also showed enrichment in glycerophospholipid metabolism and N-glycan biosynthesis, indicating potential shifts in membrane remodelling and glycoprotein synthesis. Elevated dolichyl-D-glucosyl phosphate, a key intermediate in N-glycan biosynthesis, has been reported to have a role in immune signalling and gut barrier integrity [65, 66].

To isolate the most robust markers from the globally similar metabolome of the treatment groups, we applied LASSO and sPLS-DA, validated by chemical enrichment analysis, and further metabolite selection by DIABLO. These independent methods consistently identified a selection of metabolites grouped as prenol lipids and cyclic peptides for T1, while indoles and other derivatives were selected for T2. Prenol lipids, a broad class of isoprenoid compounds defined by five-carbon isoprene units, include biologically active molecules such as fat-soluble vitamins, carotenoids, and triterpenoid saponins [67, 68]. Among these, several soyasaponins, including Soyasaponin III, saikosaponin B, and clinoposaponin X, were consistently elevated in T1. Soyasaponins, triterpenoid saponins present in soybeans (*Glycine max)* and other legumes, are bioactive compounds known for their anti-inflammatory, immunostimulatory, and antimicrobial properties [69]. Soyasaponin III has been reported to induce anti-chicken ovalbumin total-IgG and IgG1 antibody responses in mice [70], while saikosaponin b2 has been previously linked to inhibition of multidrug resistance-associated drug transporters [71]. In layer hens, dietary soyasaponins have been linked to improved lipid metabolism, immune function, and performance [72, 73]. Their enrichment in T1 suggests that ionophores may influence lipid transport and immune pathways, potentially enhancing cellular resilience.

Conversely, the consistently elevated presence of indole derivatives (5-methoxyindole, 2-oxindoles) in vaccinated broilers is likely linked to the complex interplay between tryptophan metabolism and immune responses [74, 75]. Indoles are produced from tryptophan by gut microorganisms, such as *Escherichia coli* and *Clostridium sporogenes*, through the microbial exogenous pathway [75]. These indoles play a significant role in modulating immune functions by activating receptors such as the aryl hydrocarbon receptor and pregnane X receptor in intestinal dendritic cells and other immune cells. Activation of these receptors is critical for maintaining immune balance, controlling inflammation, and preserving gut barrier integrity [75]. Additionally, 5-methoxyindole, a precursor in the biosynthesis of melatonin, may contribute to the observed immunomodulatory effects by influencing COX-2 expression and regulating inflammatory responses [76].

Integrating metabolomics with microbiome data provides valuable insights into potential host-microbe interactions. In this study, several microbial taxa, including *Mediterraneibacter* and *UBA3818 *(Ruminococcaceae), were statistically correlated with distinct metabolite profiles between treatment groups. Interestingly, *Mediterraneibacter* is linked to fibre degradation and the breakdown of sugars such as rhamnose through glycosidase activity [77, 78], while members of the Ruminococcaceae family are involved in tryptophan metabolism due to their ability to convert tryptophan into indole and related compounds [79, 80]. Certain members within this family also encode tryptophan decarboxylase, an enzyme that catalyses the conversion of tryptophan into tryptamine and other indole derivatives [81]. Consistent with these functional capacities, the increased abundance of *Mediterraneibacter* in the ionophore group coincided with enriched levels of saponin-related compounds as observed here. These compounds are known to positively influence gut integrity and immune function within the host [46], potentially playing a role in the immune response against *Eimeria* and other enteric pathogens.

Coccidiosis vaccination is known to induce an immune response that increases amino acid demand, resulting in the diversion of amino acids away from muscle accretion towards immune function and intestinal repair [82]. Within our study, the shifts associated with Ruminococcaceae *UBA3818* levels alongside the enrichment of tryptophan-derived metabolites (indoles) may indicate similar altered amino acid utilisation during immune challenge as occurred in T2. However, it is not possible to determine from the present data whether this reflects increased microbial degradation, host-driven metabolic reallocation, or a combination of both. Although we previously reported that the ionophore group had a better performance than the coccidiosis group in the same broiler cohort [15], we did not directly integrate performance with the present metabolomic analysis. Therefore, we considered any link between altered tryptophan metabolism, indole production, and growth production as speculative and requires further validation. Furthermore, the above interpretations should be considered with caution. Our observed associations are correlative and do not demonstrate direct metabolic activity in situ. Likewise, the presence of KO genes reflects function potential rather than confirmed active pathway expression under the conditions studied. Further targeted or experimental validation would be required to confirm these functional relationships.

In our metagenome study, the abundance of *B. fragilis* was significantly higher in the ionophore group than the vaccinated group [15], consistent with previous findings showing enrichment of *Bacteroides* species in broilers treated with salinomycin [10, 83]. In the current study, *B. fragilis* exhibited the highest correlation density, particularly with amino acids and glycerophospholipids, serving as a major metabolic associate of the T1 environment. However, highly connected taxa in supervised integration frameworks such as DIABLO can sometimes result from strong shared covariance with treatment structure (i.e. both taxon and metabolites track strongly with ionophore treatment), rather than direction biochemical interaction with every correlated metabolite. The observed network centrality of *B. fragilis* should be interpreted cautiously and not necessarily as evidence of functional dominance. Nevertheless, the alignment between its genomic potential and the observed metabolite enrichment is noteworthy. Genome-scale models have shown that *B. fragilis* can utilise over 140 different nutrients [84], and it has been reported to metabolise amino acids and complex carbohydrates like exopolysaccharides to optimise energy yield and maintain redox balance, especially under carbohydrate-limited conditions [85]. Interestingly, a recent metabolic modelling study demonstrated *B. fragilis* as a central metabolic hub, exerting a dominant influence on the functional organisation and metabolite exchange networks within the chicken caeca [86]. Overall, these findings suggest that *B. fragilis* could be a candidate for future functional studies, such as isotope-tracing or gnotobiotic trials, to determine whether these associations represent direct influence or shared environmental response. While its probiotic potential is supported by studies in laying hens [16], its role in broilers remains debated, with conflicting evidence on its association with feed efficiency [87, 88].

Despite the valuable insights into the metabolic effects of T1 and T2, several limitations must be considered. The most critical constraint is the substantial proportion of unclassified metabolites, which limits the depth of pathway-level interpretation and restricts our ability to fully resolve the functional consequences of microbial shifts. This reflects broader challenges in untargeted metabolomics, particularly in non-model species where incomplete reference libraries hinder accurate annotation. A second major limitation relates to the inherent uncertainty of compound identification in untargeted workflows. Although annotation was performed using high-quality spectral matching, a subset of identifications were biologically implausible, likely reflecting spectral similarity artefacts or gaps in existing databases [53, 89]. For instance, a LASSO-selected feature, C3313, was putatively annotated as fluvoxamino acid [36]. Given that this is a metabolite of a human antidepressant, its presence in a controlled broiler trial is likely a database mis-annotation of an endogenous or microbial isomer. This feature remains a significant discriminatory marker of T2, but its structural identity requires further validation beyond formula matching. These results indicate the lack of harmonised QA/QC guidelines and the risk of erroneous compound identification in untargeted workflows. Consequently, such annotations should be interpreted with caution, and future validation using authentic standards or targeted approaches is warranted. Furthermore, while differential abundance was observed for certain metabolites, their biological relevance remains unclear and requires confirmation through functional assays. Correlations between specific microbes and metabolite profiles should also be interpreted cautiously, as they represent statistical associations rather than confirmed functional interactions. In the context of a two-group study design, where both microbial taxa and metabolites co-vary strongly with treatment, correlation structures may be driven by shared group membership rather than direct biological interactions. This effect may be further amplified by the compositional nature of relative abundance data, which can inflate apparent positive correlations among features that are jointly enriched within a treatment group. Collectively, our findings should be interpreted as associative and hypothesis-generating rather than definitive, as establishing causality will require targeted metabolite validation, isotope-tracing studies, functional experimental confirmation, and integration with host immune and transcriptional datasets. These limitations highlight the need for more comprehensive chemical annotation frameworks and integrative multi-omics strategies to refine mechanistic interpretations in future research.

## Conclusion

In light of globalisation, urbanisation, and the intensification of food production systems, optimising poultry health and welfare is crucial for overall global health. Using an integrated metagenomic and metabolomic approach, this study shows that gut health strategies exert targeted rather than global effects, altering specific microbiota and metabolic pathways while leaving the overall metabolic profile largely conserved. 

In particular, ionophore treatment was linked to the enrichment of spermidine, glutathione disulfide, and prenol lipids, including soyasaponins, which are metabolites traditionally associated with anti-inflammatory and immunomodulatory effects. In contrast, vaccination using live attenuated *Eimeria* correlated with shifts in aromatic amino acid metabolism, resulting in production of indole-derivatives including 5-methoxyindole. The integration of metabolomics and microbiome data reveals complex host-microbiota interactions, with specific microbial taxa, such as *Mediterraneibacter* and Ruminococcaceae *UBA3818*, appearing as candidate drivers of these metabolic outcomes.

While these relationships are associative and require future validation through functional assays or gnotobiotic trials, they provide a mechanistic blueprint for precision coccidiosis control. From a practical perspective, these results suggest that different coccidiosis strategies may be associated with distinct gut health outcomes. These differences could be relevant when considering complementary management approaches and may potentially influence overall bird performance. While direct applications cannot be inferred from the present study, the identified signatures provide a basis for generating testable hypotheses around targeted dietary modulation. Future work could test whether supplementing specific microbial metabolites can reproduce physiological signatures observed here, or whether the associated taxa hold potential as targeted probiotic candidates. Beyond poultry, this work contributes to the broader One Health initiative, emphasising the power of microbiome-informed strategies to manage enteric diseases and promote sustainable gut health across species.

## Supplementary Information

Below is the link to the electronic supplementary material.

Supplementary Material 1

Supplementary Material 2

Supplementary Material 3

## Data Availability

The metabolomics data have been deposited in the Metabo Lights repository with the study identifier [MTBLS11445] (https://url.uk.m.mimecastprotect.com/s/C350CJQBwUoKRL4HGhKfyNXZl?domain=ebi.ac.uk).

## Code availability

The codes used in this study are available at https://github.com/gladysmariapangga/integrative_omics.

## Abbreviations

AUC: Area under the curve
BER: Balanced error rate
CER: Classification error rate
ChemOnt: Chemical Ontology
CV: Cross-validation
DIABLO: Data Integration Analysis for Biomarker Discovery using Latent Variable Approaches for Omics Studies
GSEA: Gene set enrichment analysis
IND: Indole and indole derivatives group
KEGG: Kyoto encyclopedia of genes and genomes
KO: KEGG orthology
LASSO: Least Absolute Shrinkage and Selection Operator
LC-MS: Liquid chromatography – mass spectrometry
HMDB: Human metabolome database
MAG: Metagenome assembled genome
MeSH: Medical Subject Headings
m/z: Mass-to-charge ratio
PCoA: Principal coordinate analysis
PL: Prenol lipids
PQN: Probability quotient normalisation
RT: Retention time
SCFA: Short-chain fatty acids
SMILES: Simplified Molecular Input Line Entry System
sPLS-DA: sparse Partial Least Squares Discriminant Analysis
