# Supplementary material for: Integrated metabolomics and metagenomics reveal divergent caecal metabolic signatures following commercial gut health interventions in broilers
Source: Anim Microbiome. 2026 Jul 4;8:99. doi: 10.1186/s42523-026-00596-z (PMC13621688; doi:10.1186/s42523-026-00596-z)
Supplement: Supplementary file 1 [file 42523_2026_596_MOESM1_ESM.docx]

Supplementary information

**Methods**

*Metabolite extraction*

For extraction, 25 mg of caecal contents were weighed and placed into thick centrifuge tubes along with 2 magnetic beads. Then, 10 μL of the prepared internal standard 1 was added to each sample. Next, 800 μL of a precooled extraction reagent (methanol: acetonitrile: water in a 2:2:1 ratio, v/v/v) was added, and the samples were ground for 5 minutes at 50 Hz. The ground samples were then placed at -20°C for 2 hours. After this, the samples were centrifuged at 25,000 g and 4°C for 15 minutes. Subsequently, 600 μL of each sample was transferred into new EP tubes and freeze-dried. The dried samples were reconstituted with 600 μL of 50% methanol and shaken until completely dissolved. The samples were then centrifuged again at 25,000 g and 4°C for 15 minutes. The supernatant was collected and placed in new EP tubes. Finally, 10 μL of each sample was mixed into QC samples, and the prepared supernatant was processed using LC-MS/MS.

*Ultra-performance liquid chromatography-mass spectrometry (UPLC-MS Analysis)*

A Waters UPLC I-Class Plus (Waters, USA) paired with a Q Exactive high-resolution mass spectrometer (Thermo Fisher Scientific, USA) was utilized for the separation and detection of both polar and nonpolar compounds metabolites. For nonpolar compounds, chromatographic separation was carried out using a Waters ACQUITY UPLC BEH C18 column (1.7 μm, 2.1 mm × 100 mm, Waters, USA) maintained at a temperature of 45°C. The mobile phase in positive mode consisted of 0.1% formic acid and acetonitrile, while in negative mode it was composed of 10 mM ammonium formate and acetonitrile. The gradient conditions were as follows: 0-1 min at 2% acetonitrile; 1-9 min from 2% to 98% acetonitrile; 9-12 min at 98% acetonitrile; 12-12.1 min from 98% to 2% acetonitrile; and 12.1-15 min at 2% acetonitrile. The flow rate was set to 0.35 mL/min and the injection volume was 5 μL.

Meanwhile, chromatographic separation for polar compounds was carried out on a BEH Amide column (1.7 μm, 2.1 × 100 mm, Waters, USA). In positive ion mode, mobile phase A was composed of 95% acetonitrile in water with 10 mM ammonium formate and 0.1% formic acid, while mobile phase B consisted of 50% acetonitrile in water with 10 mM ammonium formate and 0.1% formic acid. For the negative ion mode, mobile phase A was made up of 95% acetonitrile in water with 10 mM ammonium formate (adjusted to pH 9 with ammonia water), and mobile phase B consisted of 50% acetonitrile in water with 10 mM ammonium formate (also adjusted to pH 9 with ammonia water). The column temperature was maintained at 30°C. The gradient conditions were set as follows: 2% acetonitrile from 0.0-0.5 minutes, 2%-50% acetonitrile from 0.5-12.0 minutes, 50%-98% acetonitrile from 12-14 minutes, held at 98% acetonitrile from 14-16 minutes, and then washed with 2% acetonitrile from 16.1-18 minutes.

For mass spectrometry, the Q Exactive (Thermo Fisher Scientific, USA) was used for both primary and secondary data acquisition. The full scan range was 70-1050 m/z with a resolution of 70,000, and the automatic gain control (AGC) target for MS acquisitions was set to 3x10^6^ with a maximum ion injection time of 100 ms. The top three precursors were selected for subsequent MS/MS fragmentation with a maximum ion injection time of 50 ms and a resolution of 17,500, with the AGC set to 1x10^5^. The stepped normalized collision energy was set to 20, 40, and 60 eV. The ESI parameters were as follows: sheath gas flow rate at 40, auxiliary gas flow rate at 10, spray voltage of 3.80 kV in positive-ion mode and 3.20 kV in negative-ion mode, capillary temperature at 320°C, and auxiliary gas heater temperature at 350°C.

*Sparse partial least squares discriminant analysis (sPLS-DA)*

Sparse Partial Least Squares Discriminant Analysis was performed using the mixOmics R package to explore group separation based on the metabolomic dataset. The number of components for the final model was set to 10, exceeding the number of classes in the outcome variable to ensure a comprehensive representation of latent structures. To identify the optimal number of variables to retain from the predictor matrix (X), we implemented a tuning grid ranging from 20 to 200 variables in increments of 2 (i.e., keepX = seq(20, 200, 2)), using 2 components during the tuning process. Model performance was assessed using M-fold cross-validation with 5 folds and 10 repeats, applying the overall misclassification error rate as the primary criterion. The maximum distance (max.dist) metric was used for sample classification and evaluation of discriminative performance.

To visualize feature contributions, we set the loading contribution color to correspond with the group showing the maximum expression of each variable, and the contribution was assessed based on the mean abundance across groups. Ellipses were drawn around group centroids to aid in visual interpretation of separation.

These settings were chosen to balance interpretability and model robustness, particularly when dealing with high-dimensional, potentially skewed metabolomic data.

*Data Integration with DIABLO (Data Integration Analysis for Biomarker discovery using Latent variable approaches for Omics studies)*

DIABLO was performed using the mixOmics R package to explore the coordinated patterns between caecal metagenomic and metabolomic data in relation to the experimental groups. DIABLO was employed as a supervised multivariate method to integrate these two omics datasets and identify multi-omics signatures associated with the interventions.

The integration model included two blocks: the metagenome and the metabolome. A design matrix was specified to define the strength of association between datasets, with off-diagonal values set to 0.1, thereby establishing moderate links between the blocks while avoiding forced over-correlation. Diagonal elements were set to zero to prevent self-linking.

The number of latent components was set to 10, exceeding the number of experimental groups to better capture complex variance structures across the integrated datasets. Tuning was performed manually across 3 components, with a range of features retained from each block: 10 to 50 features (step 5) from the metagenomic dataset, and 50 to 500 features (step 50) from the metabolomic dataset. This tuning grid was chosen to ensure a balanced representation across both omics types while maintaining manageable dimensionality for interpretation.

Model validation was conducted using Leave-One-Out (LOO) cross-validation with 10 repeats, assessing classification performance based on the overall misclassification rate. The centroids distance (centroids.dist) metric was employed for class prediction and sample discrimination.

**Results**

**Figure S1. Classification error rate of SPLS-DA analysis where the diamond represents the number of discriminatory features selected in each component.**

**Figure S2. High-Resolution Mass Spectrometry Spectral Validation of Primary Discriminatory Metabolites.**

*Legend:* Representative MS1 and MS2 spectra for key biomarkers identified via C18 reverse-phase chromatography. Features are annotated with accurate monoisotopic mass (m/z), molecular formula, and retention time (RT). Isotopic distributions (indicated by purple bars) demonstrate fidelity to theoretical patterns.
